# Multicompare Tests of the Performance of Different Metaheuristics in EEG Dipole Source Localization

**DOI:** 10.1155/2014/524367

**Published:** 2014-03-16

**Authors:** Diana Irazú Escalona-Vargas, Ivan Lopez-Arevalo, David Gutiérrez

**Affiliations:** ^1^Information Technology Laboratory, Center for Research and Advanced Studies (Cinvestav), Ciudad Victoria, TAMPS 87130, Mexico; ^2^Biomedical Signal Processing Laboratory, Center for Research and Advanced Studies (Cinvestav), Apodaca, NL 66600, Mexico

## Abstract

We study the use of nonparametric multicompare statistical tests on the performance of simulated annealing (SA), genetic algorithm (GA), particle swarm optimization (PSO), and differential evolution (DE), when used for electroencephalographic (EEG) source localization. Such task can be posed as an optimization problem for which the referred metaheuristic methods are well suited. Hence, we evaluate the localization's performance in terms of metaheuristics' operational parameters and for a fixed number of evaluations of the objective function. In this way, we are able to link the efficiency of the metaheuristics with a common measure of computational cost. Our results did not show significant differences in the metaheuristics' performance for the case of single source localization. In case of localizing two correlated sources, we found that PSO (ring and tree topologies) and DE performed the worst, then they should not be considered in large-scale EEG source localization problems. Overall, the multicompare tests allowed to demonstrate the little effect that the selection of a particular metaheuristic and the variations in their operational parameters have in this optimization problem.

## 1. Introduction

The problem of source localization is of great interest in neuroscience. It has applications in areas such as clinical sciences and brain research [1]. Techniques based on electroencephalography (EEG) measure the electric potentials on the scalp and process them to infer the location of the underlying neural activity. Such inference is mainly based in the solution of two problems: first, the* forward* problem of computing the electric potentials over the scalp given a current source within the brain, which may be solved by selecting a proper model to approximate the volume conductor, in addition to the dipole model already assumed for the source signals; second, the* inverse* problem of finding the current distributions using EEG measurements, which often involves an iterative solution of the forward problem until an optimization criterion is attained. Therefore, it is important to have efficient optimization algorithms in order to solve the inverse problem based on this modeling-optimization approach.

The forward problem delivers an objective function which usually is very complex and has many local optima, especially when the number of dipole sources is large, or when dealing with low signal-to-noise ratio (SNR) conditions. Hence, metaheuristic techniques are promising candidates to help solving this problem as they are designed to escape from local optima and proceed with the exploration of the search space until finding the global optima in modest computation times. The most representative algorithms are simulated annealing (SA), genetic algorithms (GA), particle swarm optimization (PSO), differential evolution (DE), and tabu search (TS). TS and SA (referred to as trajectory methods) work on one or several neighborhood structures imposed on the solutions of the search space. Evolutionary techniques, such as GA, PSO, and DE, incorporate a learning component in the sense that they implicitly or explicitly learn correlations between decision variables to identify high quality areas in the search space. Evolutionary metaheuristics perform a biased sampling of the search space by recombination of solutions.

Comparative studies of metaheuristics for solving the inverse problem have been performed in the past, and they are provided with valuable information regarding the performance of the methods in the localization of one or multiple dipoles. Some examples of such studies are given next: In [2], Lewis and Mosher proposed GA as a promising approach to find minimal source solutions using distributed dipoles modeling in magnetoencephalography (MEG) signals. In [3], Haneishi et al. considered SA in the localization of multiple dipoles using noiseless MEG data, and they found that SA is effective in solving the inverse problem, but it had a high computational performance. Moreover, they detailed the implementation of the SA algorithm in [4], where a modification of the method for its implementation in parallel computers was proposed. In [5], Gerson et al. performed a comparative study between the SA and simplex method using EEG data under noiseless conditions, in which they determined the sensitivity of the methods through simulations under different initial assumption of the dipole's position. They concluded that the simplex algorithm was affected with the different initial solutions whereas the SA performance was not affected. Furthermore, the simplex method delivered large errors even when they proposed solutions near to the global optimum, and forfeited its convergence speed advantage compared with SA. In [6], McNay et al. used GA for the estimation of two dipoles using EEG signals under specific noise conditions. They also studied the localization accuracy of the algorithm using a physical model incorporating potential measurements of two simultaneously active sources embedded in a sphere. In [7], Khosla et al. compared SA and simplex method in localizing three dipoles in EEG data under noise conditions. They used different SA's parameter settings from Gerson et al. [5], and also they concluded that SA is a feasible method even if it does not provide a good solution from the beginning. In [8], Uutela et al. compared a clustering method, GA, and SA for localizing two dipoles using MEG signals under noiseless conditions. GA was the most effective method whereas the clustering method performed well when the number of sources was small. In [9], Nagano et al. used GA with MEG recordings when localizing two dipoles under various noise conditions. The authors concluded that GA was a robust method for high SNR conditions. In [10], Scherg et al. reconstructed multiple sources using EEG/MEG data simultaneously acquired and through GA with sequential dipole fitting strategy. In [11], Jiang et al. compared GA, SA, and TS methods for localizing three dipoles using MEG data. They found that the three algorithms converged to the global optimum if the computational resources are unlimited, but the best results were achieved with a hybrid GA under noise conditions. In [12], Zou et al. performed a comparative study between a hybrid GA and simplex using EEG data. They found that the hybrid GA gives better solutions under specific noise conditions. In [13], Li et al. implemented a DE algorithm in the localization of multiple dipoles under noiseless conditions. They concluded that DE is a feasible metaheuristic in the reconstruction of EEG source localization with single dipole sources but not when there are two or more dipoles active at the same time. In [14], Sequeira et al. performed GA and its hybrid version using MEG data in the localization of deep and cortical sources. There, the authors used two-objective functions to make the algorithm more precise and efficient in terms of computation times. They found that GA was a robust method to determinate the positions of multidipole sources simultaneously. In [15], Qiu et al. compared PSO and GA for high SNR conditions. They found that the PSO algorithm was more accurate and required less computational effort than GA in EEG source localization. In [16], Jiang et al. used GA to perform a rough source location and then applied the music (multiple signal classification) method to refine the search until obtaining an accurate position of MEG sources. They concluded that the GA-music strategy improved the speed and accuracy in source localization under noiseless conditions. Furthermore, they obtained same results when the PSO algorithm was used instead of GA [17], but the PSO-music approach was a better strategy than GA-music. In [18], Alp et al. used PSO algorithm in the localization of the sources of event related potentials (ERP). The authors concluded that PSO was accurate even when the ERP sources generated signals that overlap in time and frequency. In [19], Parsopoulos et al. compared PSO and the unified PSO (UPSO) in MEG source localization. They found that UPSO exhibited better efficiency and robustness in the case of MEG noiseless data, and it seemed to be less affected by relatively small increases in the number of sensors than PSO. Regardless of noise conditions, the efficiency of both algorithms was similar. In [20], Rytsar and Pun compared SA and GA using EEG data. The best results were achieved with GA, but its computational cost was higher compared with SA algorithm. In [21], Shirvany et al. proposed a new global optimization method based on PSO to solve EEG source localization under noiseless conditions. They concluded that the new strategy of PSO found the optimal solution faster than other PSO methods from the literature, and it was less prone to be trapped in local minima.

Metaheuristics are often compared in terms of CPU times, solution quality, parallel speedups, and function evaluation counts whereas most of the studies in solving the EEG/MEG inverse problem evaluate the performance of the metaheuristics in terms of the mean squared error (MSE) under very specific SNR conditions. In our previous work (see [22]), we performed a comparative study of SA, GA, PSO, and DE under realistic conditions and for different values of their operational parameters. We used the concentrated likelihood function (CLF) as objective function and the Cramér-Rao bound (CRB) as a generalized lower-bound to compare the efficiency of the metaheuristics, rather than concentrating only on the MSE. In the case of SA, GA, and PSO methods, we used the operational parameters that have proved to work well in the EEG inverse problem. However, for the case of DE we performed an exhaustive simulation to find a combination of parameters for which an optimal solution was attained in the localization of two correlated dipoles. Our results showed that the performance of SA, GA, PSO, and DE decayed as the noise increased, while SA and PSO seemed to be very sensitive to the correlation between the sources. Overall, GA was the most attractive technique in terms of performance using the CRB as a reference. However, a formal statistical analysis was not performed in order to show if the differences in the performance between metaheuristics were indeed statistically significant. Hence, in this paper we propose a multicompare analysis of variance (ANOVA) in order to evaluate such differences.

This paper is organized as follows: in Section 2, we pose the source localization problem as the optimization of the concentrated likelihood function (CLF); in Section 3, we describe the multicompare tests by which we evaluate the performance of the metaheuristics; in Section 4, we present the experimental settings when using simulated EEG data; in Section 5, the results of our numerical examples are shown; in Section 6, we give our concluding remarks and future work.

## 2. The Optimization Problem

The inverse problem may be solved by modeling the source signal and the volume conductor in the following way [23].

- A model is proposed for the source signal. In our case, we will use current dipoles, which are widely used to approximate the brain activity in evoked response and event related experiments [24].
- A model is constructed for the volume conductor. The accuracy of the conductor model must be as good as or better than that of the source model. Here, we assume the classical concentric four sphere model to approximate the head geometry. This model is justified for sources near the surface [25].
- At least as many independent EEG measurements are made as the model has independent variables. Under those conditions, the mathematical representation of the problem would have as many equations as unknowns, and the variables of the model could be evaluated.

Based on this idea, it is possible to iteratively solve the inverse problem by assuming known values for the variables of the model and then solving the forward problem for those assumed parameters and finally comparing the computed EEG against the measured data. Then, the process of solving the inverse problem becomes an* optimization* problem if we define an objective function and, at each iteration, the assumed parameters are adjusted and the objective function is reevaluated until an optimality criterion is attained. Next, we go into further detail about defining the concentrated likelihood function, which will be the objective function to be used throughout this paper.

Let us consider that a source of brain activity is modeled as a single dipole with a moment **q** ∈ ℝ^3^, which is located at position **r**_*q*_ ∈ ℝ^3^ within the brain. For the *m*th-sensor located on the scalp at **r**_*m*_ ∈ ℝ^3^, *m* = 1,…, *M*, the surface potential can be expressed as *v*_*m*_ = *g*_*m*_^*T*^(***θ***)**q**, where *g*_*m*_^*T*^(***θ***) is the gain vector (or* kernel* vector) which is a function of the vector of parameters ***θ***. Under these conditions, we can define a potential vector as

(1) $$\begin{array}{c} v={\left[v_{1},v_{2},\ldots ,v_{M}\right]}^{T}=A\left(\theta \right)q, \end{array}$$

where *A*(***θ***) is the *M* × 3* lead-field* matrix, in which the *m*th-row corresponds to *g*_*m*_^*T*^(***θ***). *A*(***θ***) is derived from using the quasistatic approximation of Maxwell's equations on a volume that approximates the head's geometry. In a physical sense, *A*(***θ***) represents the material and geometrical properties of the medium in which the sources are submerged. Furthermore, the model in (1) can be extended to a spatiotemporal representation by allowing change in time. Hence, if we assume that the source remains at the same position during the measurement period, we obtain the following:

(2) $$\begin{array}{c} v\left(t\right)=A\left(\theta \right)q\left(t\right), \end{array}$$

for *t* = 1,2,…, *N* time samples. Finally, in the case of *p* distinct dipoles, (2) holds with **q**(*t*) and *A*(***θ***) substituted with **q**(*t*) = [**q**_1_(*t*),**q**_2_(*t*),…,**q**_*p*_(*t*)]^*T*^, and *A*(***θ***) = [*A*_1_(***θ***), *A*_2_(***θ***),…, *A*_*p*_(***θ***)], respectively.

Equation (2) can be used to represent the forward model as a linear measurement model in the presence of additive noise as follows:

(3) $$\begin{array}{c} Y_{k}\left(t\right)=v\left(t\right)+E_{k}, \end{array}$$

where *Y*_*k*_(*t*) is the matrix of measurements obtained from *k* = 1,2,…, *K* independent experiments, and *E*_*k*_ is the matrix of noise. Under these conditions, our goal is to determine ***θ*** = **r**_*q*_ from (3) that best describes the EEG measurements. Here, we consider the maximum likelihood (ML) technique to estimate the position parameters, as it has been shown that an unbiased estimate of ***θ*** (denoted as $\hat{\theta }$) can be obtained through the following optimization problem [26]:

(4) $$\begin{array}{c} \hat{\theta }=\underset{\begin{array}{c} \;\;\theta \; \end{array}}{\min}\;F\left(\theta \right), \end{array}$$

where *F*(***θ***) is the concentrated likelihood function (CLF), defined as

(5) $$\begin{array}{c} F\left(\theta \right)=\mathrm{tr}\left\{\left(I-A{\left(A^{T}A\right)}^{-1}A\right)R\right\}, \end{array}$$

where tr⁡{·} is the trace operator, *I* is the identity matrix, *A* = *A*(***θ***) is used for simplicity in the notation, and *R* is the data's covariance matrix. When unknown, a consistent estimate of *R* is usually obtained as

(6) $$\begin{array}{c} \hat{R}=\frac{1}{N}\sum_{t=1}^{N}Y\left(t\right)Y{\left(t\right)}^{T}. \end{array}$$

Therefore, the CLF corresponds to the objective function in the optimization problem from which the dipole parameters will be estimated. In our case, ***θ*** can be estimated by minimizing (5) through the proposed metaheuristics. For that matter, let us consider that *Ω*_brain_ is the brain's domain where the dipole's location is determined. Then, we pose the following optimization problem:

(7) θ^  =min⁡⁡θ  ∈Ωbrainb≤θ≤aF(θ),

where *a* and *b* are constant constraints.

Given that the CLF is derived from ML principles, the resulting estimates have a handful of desired properties: they are consistent, asymptotically Gaussian distributed, and asymptotically efficient [27]. Thus, the optimization is expected to converge to the true value for a sufficiently large number of data samples (i.e., *KN* ≫ *M*). In such a case, the bias in the estimation disappears asymptotically and the variance approaches zero. Moreover, no other bias-free estimator exists with a smaller variance. Then, now the question is how to determine the best suited metaheuristic to solve the problem in (7), for which we propose the multicomparison tests described next.

## 3. Multicompare Tests

Statistical analysis is a powerful tool to evaluate the performance of algorithms, as well as to quantify the relationship between algorithm performance and other factors describing problem characteristics. We find in the literature many methods for such purposes: the analysis of variance (ANOVA),* t*-test,* F*-test, and least-squares regression, as well as robust alternatives such as Friedman's test and L1-regression. Pairwise comparisons are the simplest type of statistical tests used to compare the performance of two algorithms in a common set of problems. In multiproblem analysis, a value for each pair of algorithm/problem is required. However, when there are more than two groups, a multicompare approach is needed.

Multiple comparisons of various algorithms must be carried out by first using a statistical method for testing the differences among the related samples means. Once this test rejects the hypothesis of equivalence of means, the detection of the concrete differences among the algorithms can be done with the application of a* post hoc* statistical process. Parametric tests have been commonly used in the analysis of experiments in computational intelligence. Unfortunately, they are based on independence, normality, and heteroscedasticity assumptions, which are most probably not attained when analyzing the performance of stochastic algorithms based on computational intelligence [28]. Nonparametric tests are used to overcome this problem as they do not need prior assumptions related to the sample of data to be analyzed. Furthermore, nonparametric tests have been already used for comparing metaheuristic algorithms in several benchmark functions [29].

In our case, we use the Kruskal-Wallis test, which compares the medians between two or more samples in order to determine if they originate from the same distribution [30]. The Kruskal-Wallis test is used to compare groups when the distribution does not prove prove to be normal or when their variances are different (this latest condition applies to the problem of EEG source localization, as demonstrated in [22]). In our case, the null hypothesis is considered as *H*_0_ *: Each metaheuristic has the same median performance*. Then, when the Kruskal-Wallis test leads to significant results, it would indicate that at least one median performance is different from another.

Under these conditions, the analysis of the performance is conducted as follows.

(1) Metaheuristics under different operational parameters are used in the estimation of dipole's position by solving (7) for a fixed number of evaluations of (5);
(2) the optimization process is repeated 100 times under independent noise conditions;
(3) at each trial, the MSE is computed as
  (8) $$\begin{array}{c} \text{MSE}=\sqrt{{\left(\theta_{x}-{\hat{\theta }}_{x}\right)}^{2}+{\left(\theta_{y}-{\hat{\theta }}_{y}\right)}^{2}+{\left(\theta_{z}-{\hat{\theta }}_{z}\right)}^{2}}, \end{array}$$
 where ***θ*** = [*θ*_*x*_,*θ*_*y*_,*θ*_*z*_]^*T*^ and $\;\hat{\theta }={\left[{\hat{\theta }}_{x},{\hat{\theta }}_{y},{\hat{\theta }}_{z}\right]}^{T}$ are the true and estimated Cartesian coordinates of the dipole's position, respectively;
(4) The MSE values are used to perform the Kruskal-Wallis test and, if it reveals that at least one median performance is different, then the Dunn-Sidak* post hoc* test is performed to identify pairs of metaheuristics with significant different performances. The Dunn-Sidak test determines the critical values to reject the null-hypothesis as follows:
  (9) $$\begin{array}{c} \alpha_{i}=1-{\left(1-\alpha_{e}\right)}^{1/n}, \end{array}$$
 for *i* = 1,2,…, *n* groups being compared, *α*_*i*_ is the significance level of each individual test, and *α*_*e*_ is the family-wise or experiment-wise significance level [31].

## 4. Experimental Settings

**Figure 1:**
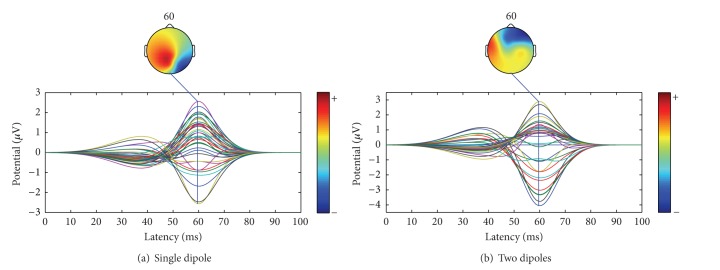
Simulated EEG data under noiseless conditions in a spherical head model.

We generated EEG data which simulated a typical evoked responses [32] (see Figure 1) for one and two correlated dipole sources using the classical spherical head model with an array of *M* = 37 electrodes. The multishell spherical head model includes four concentric layers for the brain, cerebrospinal fluid (CSF), skull, and scalp. The radii for each of the layers were, respectively, 92.45, 89.29, 85.10, and 83 mm. These layers were considered to be isotropic and to have homogeneous conductivities of 0.33, 0.0042, 1, and 0.33 S/m, respectively. Those values of radii and conductivities were chosen in accordance to the model proposed in [25]. Next, we added uncorrelated random noise to obtain SNR = 0 dB, and SA, GA, PSO, and DE methods were used in the estimation of dipole's position using the parameter settings defined in [22]. The stopping criterion of all metaheuristics was set up for a number of function evaluations of 1000 and 2500 when estimating one and two correlated dipoles, respectively. In all experiments, a PC Intel Xeon E3 quad-core at 3.10 GHz with 8 GB in RAM was used. Then, at each trial we calculated the MSE defined in (8), and those values were used to perform the multicompare test.

In order to simplify the optimization process, we translated the dipole's position to the spherical coordinates *ϑ*, *φ*, and *ϱ*, which correspond to the azimuth angle, elevation, and eccentricity, respectively. Then, the optimization problem was solved as a function of *ϑ* and *φ* only, while the eccentricity was kept to a fixed value of *ϱ* = 83 mm. Therefore, *a* and *b* in (7) corresponded to the lower- and upper-bound constraints of *ϑ* and *φ*. In our experiments, the single dipole was located at *ϑ* = 0.5235 rad and *φ* = −1.2 rad (see Figure 1(a)). For the case of two correlated dipoles, they were located at *ϑ*_1_ = *ϑ*_2_ = 0.5235 rad, while their azimuth angles were *φ*_1_ = −0.6 rad and *φ*_2_ = 0.6 rad, respectively, (see Figure 1(b)).

## 5. Results

In this section we present the results of our multicompare tests of the performance.

### 5.1. Single Dipole

**Figure 2:**
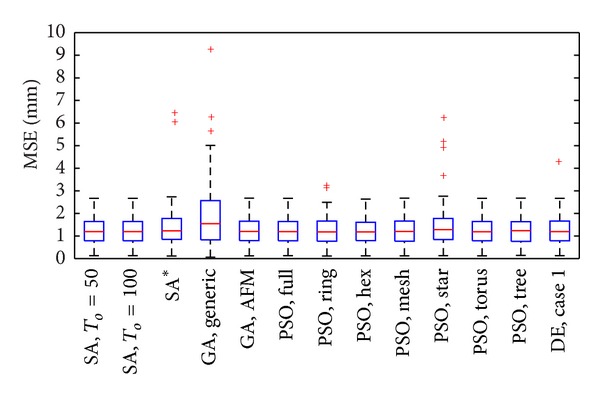
MSE of $\;\hat{\theta }$ in one-dipole localization using SA, GA, PSO, and DE with different initialization parameters, 1000 evaluations of the objective function and SNR = 0 dB. SA* correspond to the case of SA when $T_{o}=\Delta \overset{-}{E}/\ln(\beta^{-1})$. AFM refers to the case when adaptive feasible mutation was used for GA. For the PSO algorithm, *c*_1_ = *c*_2_ = 2.83 was used in all cases. DE Case 1 corresponds to the experiment using the generic strategy (DE/rand/1/bin) with Γ = *ζ* = 0.9. The central mark in each method corresponds to its median, the edges of the box are the 25th and 75th percentiles, the whiskers extensions are the most extreme data points, and “+” markers correspond to the outliers.

The problem of estimating the location of a single dipole in our settings corresponded to find $\;\hat{\theta }=[\hat{\vartheta },\hat{\varphi }]$. Since thirteen different operational parameters were varied among the four metaheuristics evaluated (see [22] for further detail), here we used box plots to show the results of the evaluation of the performance. There, the MSE is given in millimeters. Hence, in Figure 2 we can observe that the median MSE of the generic GA is slightly different in comparison to the other metaheuristics, but the statistical test did not reveal significant differences in the performance at a significance level of 0.01.

### 5.2. Two Correlated Dipoles

**Figure 3:**
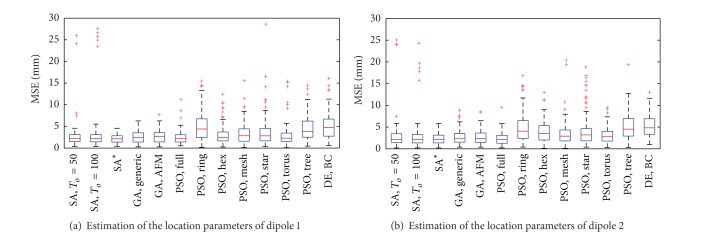
MSE of $\;\hat{\theta }$ in two-dipole localization using SA, GA, PSO, and DE with different initialization parameters, 2500 evaluations of the objective function and SNR = 0 dB. SA* correspond to the case of SA when $T_{o}=\Delta \overset{-}{E}/\ln(\beta^{-1})$. AFM refers to the case when adaptive feasible mutation was used for GA. For the PSO algorithm, *c*_1_ = *c*_2_ = 2.83 was used in all cases. DE BC corresponds to the experiment using the generic strategy (DE/rand/1/bin) with Γ = 0.5 and *ζ* = 0.2. The central mark in each method corresponds to its median, the edges of the box are the 25th and 75th percentiles, the whiskers extensions are the most extreme data points, and “+” markers correspond to the outliers.

In this case, the optimization process corresponded to find $\;\hat{\theta }=[{\hat{\vartheta }}_{1},{\hat{\varphi }}_{1},{\hat{\vartheta }}_{2},{\hat{\varphi }}_{2}]$.  Figure 3 shows the results of the evaluation of the performance. There, we can observe that the algorithms achieved different results. Therefore, a summary of the results of the Dunn-Sidak tests are shown in Table 1, where “√” and “×” indicate if the corresponding metaheuristics had indeed different performances or not, respectively. Note that the family-wise and individual significance levels were, respectively, *α*_*e*_ = 0.01 and *α*_*i*_ = 1 − (1 − 0.01)^1/*n*^, where $n=\left(\begin{array}{c} 13\\ 2 \end{array}\right)=78$. The values that are shown below the metaheuristic's name (denoted as $\;{\tilde{\theta }}_{1}$) correspond to the median of the MSE evaluated over the *K* = 100 independent trials. Note that we only show the multicompare results corresponding to one of the two dipoles' location as the errors for the other dipole that are estimated with the same metaheuristic were very similar. We can observe from Table 1 that the most viable method was the SA with $T_{o}=\Delta \overset{-}{E}/\ln(\beta^{-1})$ as it obtained a minimum median error (MSE = 2.15) but, as the analysis in [22] demonstrated, this method had bad performance under low SNR conditions for the case of two correlated dipoles. Therefore, the analysis that is proposed here and the one in [22] are complementary and should be performed together in order to fully evaluate different optimization methods.

## 6. Conclusions

In this paper we used nonparametric multicompare tests to evaluate the performance of different metaheuristics in solving an optimization problem for EEG dipole source localization. We evaluated the performance in terms of metaheuristics' operational parameters and for a fixed number of evaluations of the objective function. Through this process, we were able to link the metaheuristics' efficiencies with a common measure of computational cost. The results showed that there were no significant differences between the SA, GA, PSO, and DE in one-dipole estimation. For two correlated dipoles, the SA algorithm with $T_{o}=\Delta \overset{-}{E}/\ln(\beta^{-1})$ was the most viable method, while the worst performing methods were PSO (ring and tree topologies) and DE. However, the performance of SA is very sensitive to other factors, such as the SNR conditions. Hence, the statistical analysis that is proposed here provides valuable information in terms of the bias of the estimation among different metaheuristics, but a complementary analysis is necessary in order to also evaluate the different methods' robustness.

Our proposed analysis can be easily extended to evaluate other factors, for example, the electrical properties of the surrounding tissues. It is well know that errors are introduced in the solution of the EEG inverse problem due to dissimilarities in the values of conductivities of the tissues among individuals [33–35], then it might be valuable to add those parameters in the optimization framework and evaluate the performance of metaheuristics using both the nonparametric statistical tests that are presented here and the stochastic Cramér-Rao bounds [36] in order to account also for noise effects.

Since adding the value of the conductivities as parameters in the optimization implies a more complex optimization problem, our future work will be dedicated to evaluate advanced versions of current optimization algorithms, such as steady state genetic algorithm (SSGA) [37], adaptive differential evolution (JDE) [38], parameter adaptive differential evolution (JADE) [39], and self-adaptive differential evolution (SADE) [40]. It is also important to explore new strategies such as the backtracking search optimization algorithm (BSA) [41] or the covariance matrix adaptation evolution strategy (G-CMA-ES) [42].

## Figures and Tables

**Table 1:** Multicomparative table using Kruskal-Wallis and Dunn-Sidak post hoc test in two-dipole localization using SA, GA, PSO, and DE with different initialization parameters, 2500 evaluations of the objective function, and SNR = 0 dB. SA* corresponds to the case of SA when $T_{o}=\Delta \overset{-}{E}/\ln\left(\beta^{-1}\right)$. AFM refers to the case when adaptive feasible mutation was used for GA. For the PSO algorithm, *c*_1_ = *c*_2_ = 2.83 was used in all cases. DE BC corresponds to the experiment using the generic strategy (DE/rand/1/bin) with Γ = 0.5 and *ζ* = 0.2. ${\tilde{\theta }}_{1}$ values correspond to the median of the MSE evaluated over the *K* = 100 independent trials.

	SA, *T*_*o*_ = 100 ${\tilde{\theta }}_{1}=2.16$	SA* ${\tilde{\theta }}_{1}=2.15$	GA, generic ${\tilde{\theta }}_{1}=2.43$	GA, AFM ${\tilde{\theta }}_{1}=2.65$	PSO, full ${\tilde{\theta }}_{1}=2.18$	PSO, ring ${\tilde{\theta }}_{1}=4.39$	PSO, hex ${\tilde{\theta }}_{1}=2.39$	PSO, mesh ${\tilde{\theta }}_{1}=2.85$	PSO, star ${\tilde{\theta }}_{1}=2.81$	PSO, torus ${\tilde{\theta }}_{1}=2.28$	PSO, tree ${\tilde{\theta }}_{1}=3.83$	DE, BC ${\tilde{\theta }}_{1}=4.74$
SA, *T*_*o*_ = 50 ${\tilde{\theta }}_{1}=2.19$	×	×	×	×	×	√	×	×	×	×	√	√

SA, *T*_*o*_ = 100 ${\tilde{\theta }}_{1}=2.16$		×	×	×	×	√	×	×	×	×	√	√

SA* ${\tilde{\theta }}_{1}=2.15$			×	×	×	√	×	×	×	×	√	√

GA, generic ${\tilde{\theta }}_{1}=2.43$				×	×	√	×	×	×	×	√	√

GA, AFM ${\tilde{\theta }}_{1}=2.65$					×	√	×	×	×	×	√	√

PSO, full ${\tilde{\theta }}_{1}=2.18$						√	×	×	×	×	√	√

PSO, ring ${\tilde{\theta }}_{1}=4.39$							√	×	×	√	×	×

PSO, hex ${\tilde{\theta }}_{1}=2.39$								×	×	×	√	√

PSO, mesh ${\tilde{\theta }}_{1}=2.85$									×	×	×	√

PSO, star ${\tilde{\theta }}_{1}=2.81$										×	×	√

PSO, torus ${\tilde{\theta }}_{1}=2.28$											√	√

PSO, tree ${\tilde{\theta }}_{1}=3.83$												×
